# Active Surveillance of Antimicrobial Resistance and Carbapenemase-Encoding Genes According to Sites of Care and Age Groups in Mexico: Results from the INVIFAR Network

**DOI:** 10.3390/pathogens12091144

**Published:** 2023-09-07

**Authors:** Fabian Rojas-Larios, Bernardo Alfonso Martínez-Guerra, Luis Esaú López-Jácome, Enrique Bolado-Martínez, María del Rosario Vázquez-Larios, María del Consuelo Velázquez-Acosta, Daniel Romero-Romero, Christian Daniel Mireles-Dávalos, Sandra Quintana-Ponce, José Manuel Feliciano-Guzmán, José Miguel Pérez-Hernandez, Yoselin Paola Correa-León, Eduardo López-Gutiérrez, Eduardo Rodriguez-Noriega, Esteban González-Díaz, Elena Victoria Choy-Chang, Juan Pablo Mena-Ramírez, Víctor Antonio Monroy-Colín, Alfredo Ponce-de-León-Garduño, Margarita Alcaraz-Espejel, Laura Karina Avilés-Benítez, Luís Javier Quintanilla-Cazares, Eloisa Ramírez-Alanís, Juan Manuel Barajas-Magallón, Cecilia Padilla-Ibarra, Maria Bertha Ballesteros-Silva, Noe Antonio Atanacio-Sixto, Cecilia Teresita Morales-de-la-Peña, Mario Galindo-Méndez, Talía Pérez-Vicelis, Guillermo Jacobo-Baca, Martha Irene Moreno-Méndez, María de la Luz Mora-Pacheco, Maricruz Gutiérrez-Brito, Xochitl Yadira Sánchez-Godínez, Norberta Vianey Navarro-Vargas, Luz Elena Mercado-Bravo, Alejandro Delgado-Barrientos, María Asunción Santiago-Calderón, Ismelda López-Ovilla, Alejandro Molina-Chavarria, Joaquín Rincón-Zuno, Rafael Franco-Cendejas, Sandra Miranda-Mauricio, Isabel Cristina Márquez-Avalos, Maribel López-García, Lizbeth Soraya Duarte-Miranda, Carlos Miguel Cetina-Umaña, Irma Elena Barroso-Herrera-y-Cairo, Laura Isabel López-Moreno, Elvira Garza-González

**Affiliations:** 1 Laboratorio de Microbiología, Hospital Regional Universitario de Colima, Colima 28040, Mexico; frojas@ucol.mx (F.R.-L.); noeanto2@hotmail.com (N.A.A.-S.); 2 Departamento de Infectología, Instituto Nacional de Ciencias Médicas y Nutrición Salvador Zubirán, Ciudad de México 14080, Mexico; beramg@gmail.com (B.A.M.-G.); alf.poncedeleon@gmail.com (A.P.-d.-L.-G.); 3 Servicio de Infectología, Instituto Nacional de Rehabilitación Luis Guillermo Ibarra Ibarra, Ciudad de México 14389, Mexico; esaulopezjacome@gmail.com (L.E.L.-J.); raffcend@yahoo.com (R.F.-C.); 4 Departamento de Ciencias Químico-Biológicas, Universidad de Sonora, Hermosillo 83000, Mexico; enrique.bolado@unison.mx; 5 Laboratorio de Microbiología, Servicio de Infectología y Microbiología Cínica, Instituto Nacional de Cardiología Ignacio Chávez, Mexico City 14080, Mexico; rosariovazquez_larios@prodigy.net.mx; 6 Laboratorio Clínico, Instituto Nacional de Cancerología, Mexico City 14080, Mexico; consueve62@yahoo.com.mx; 7 Laboratorios KOCH, Toluca 50214, Mexico; qfb_dano@hotmail.com; 8 Laboratorio de Microbiología Clínica, Instituto Nacional de Enfermedades Respiratorias Ismael Cosío Villegas, Mexico City 14080, Mexico; cdmirelesd@hotmail.com; 9 Facultad de Ciencias Naturales, Universidad Autónoma de Guerrero, Chilpancingo 39000, Mexico; squintanap@uagrovirtual.mx; 10 Hospital de Especialidades Pediátricas, Tuxtla Gutiérrez 29070, Mexico; jmfelguz12@gmail.com; 11 Departamento de Bioquímica y Medicina Molecular, Facultad de Medicina, Universidad Autónoma de Nuevo León, Monterrey 66460, Mexico; jmph141298@gmail.com (J.M.P.-H.); paola.correa2310@gmail.com (Y.P.C.-L.); 12 Área de Microbiología, Laboratorio Clínico, Hospital Regional de alta Especialidad de Oaxaca, Oaxaca 71256, Mexico; campos.5488@gmail.com; 13 Instituto de Patología Infecciosa y Experimental, Centro Universitario Ciencias de la Salud, Universidad de Guadalajara, Guadalajara 44280, Mexico; idfcolima@yahoo.com (E.R.-N.); doc.glzdiaz@gmail.com (E.G.-D.); 14 Departamento de Medicina Preventiva, Hospital Civil de Guadalajara, Fray Antonio Alcalde, Guadalajara 44280, Mexico; 15 Departamento de Bacteriología, Hospital General de Zona No.1 IMSS “Nueva Frontera”, Tapachula 30767, Mexico; victoriachoy21@gmail.com; 16 Laboratorio de Microbiología, Hospital General de Zona No. 21 IMSS, Centro Universitario de los Altos (CUALTOS), Universidad de Guadalajara, Tepatitlán de Morelos 47630, Mexico; dr.juanmena@gmail.com; 17 Centenario Hospital Miguel Hidalgo, Aguascalientes 20259, Mexico; vmonroyc@gmail.com; 18 Laboratorio Futura Médica, Morelia 58000, Mexico; maguii.ales@gmail.com; 19 Laboratorio de Microbiología y Parasitología, Hospital Infantil de Morelia “Eva Sámano de López Mateos”, Morelia 58253, Mexico; karisavilb@hotmail.com; 20 Laboratorio de Microbiología, Hospital Ángeles Valle Oriente, San Pedro Garza García 66260, Mexico; luisjavier_45@hotmail.com; 21 Hospital la Luz, Michoacan 58230, Mexico; sanatoriomicroera@gmail.com; 22 Servicio de Microbiología Clínica, Laboratorio DIPROMI, Morelia 58260, Mexico; qfbjmbarajas@hotmail.com; 23 Laboratorio Clínico, Hospital General de Estado “Dr. Ernesto Ramos Bours”, Hermosillo 83000, Mexico; ceciliapadillaibarra@gmail.com; 24 Centro de Diagnóstico Microbiológico S.A. de C.V, Morelia 58228, Mexico; bertha.ballesteros@cedimi.com; 25 Hospital Gral. Juan María de Salvatierra, La Paz 23080, Mexico; cecilia.mo.pe70@gmail.com; 26 Área de Microbiología, Laboratorios Galindo SC, Oaxaca 68000, Mexico; magalindom@hotmail.com; 27 Hospital Regional de Alta Especialidad Bicentenario de la Independencia, Tultitlán 54916, Mexico; 28 Centro Universitario de Salud, Universidad Autónoma de Nuevo León, Monterrey 66460, Mexico; jorge_mil@hotmail.com; 29 Laboratorios del Centro, Zamora de Hidalgo 59600, Mexico; marthai@labcen.mx; 30 Laboratorio Clínico, Hospital Ángeles Morelia, Morelia 58350, Mexico; luzmora_p@outlook.com; 31 Departamento de Epidemiología, Hospital para el Niño Poblano, Puebla 72190, Mexico; maricruz.gutierrez1910@gmail.com; 32 Laboratorio de Microbiología, Hospital Dr. Manuel Gea González, Mexico City 14080, Mexico; qfbxochitl@gmail.com; 33 Hospital General Presidente Lázaro Cárdenas del Rio, Chihuahua 31203, Mexico; norbertanavarro033@gmail.com; 34 Hospital General Dr. Miguel Silva, Morelia 58300, Mexico; lucylu.lyad@gmail.com; 35 Hospital General de León, León 37670, Mexico; alejandrodelgadob@gmail.com; 36 Departamento de Microbiología, Hospital General de Zona No. 1 IMSS Oaxaca, Oaxaca 68000, Mexico; marycalderonmx@gmail.com; 37 Hospital Chiapas Nos Une Dr. Jesús Gilberto Gómez Maza, Tuxtla Gutiérrez 29045, Mexico; isloov@hotmail.com; 38 Centro Médico Dr. Ignacio Chávez, Hermosillo 83000, Mexico; thesaint_alex@hotmail.com; 39 Instituto Materno Infantil del Estado de México, Toluca de Lerdo 50170, Mexico; rinconzj@gmail.com; 40 Hospital Lic. Adolfo López Mateos, Obregón 85000, Mexico; sandra.mimau@outlook.com; 41 Departamento de Bacteriología, Hospital del Niño “Dr. Federico Gómez Santos”, Saltillo 25253, Mexico; isabelcristina@live.com.mx; 42 Hospital de la Madre y el Niño Guerrerense, Chilpancingo de los Bravo 39075, Mexico; maribel.lopez69@yahoo.com.mx; 43 Centro Integral de Atención a la Salud de ISSSTESON, Hermosillo 83200, Mexico; lizbeth.duartem@gmail.com; 44 Hospital Materno Infantil Morelos, Chetumal 77037, Mexico; carlos31952@outlook.es; 45 Laboratorio Clínico, Hospital Dr. Fernando Ocaranza, Hermosillo 83287, Mexico; irmaoseahello@yahoo.com; 46 Laboratorio Clínico, Hospital Galenia, Cancún 77504, Mexico; jlaboratorio@hospitalgalenia.com

**Keywords:** INVIFAR, carbapenem-resistance, antimicrobial resistance, MRSA, VRE

## Abstract

We analyzed the antimicrobial resistance (AMR) data of 6519 clinical isolates of *Escherichia coli* (*n* = 3985), *Klebsiella pneumoniae* (*n* = 775), *Acinetobacter baumannii* (*n* = 163), *Pseudomonas aeruginosa* (*n* = 781), *Enterococcus faecium* (*n* = 124), and *Staphylococcus aureus* (*n* = 691) from 43 centers in Mexico. AMR assays were performed using commercial microdilution systems (37/43) and the disk diffusion susceptibility method (6/43). The presence of carbapenemase-encoding genes was assessed using PCR. Data from centers regarding site of care, patient age, and clinical specimen were collected. According to the site of care, the highest AMR was observed in *E. coli*, *K. pneumoniae*, and *P. aeruginosa* isolates from ICU patients. In contrast, in *A. baumannii*, higher AMR was observed in isolates from hospitalized non-ICU patients. According to age group, the highest AMR was observed in the ≥60 years age group for *E. coli*, *E. faecium*, and *S. aureus*, and in the 19–59 years age group for *A. baumannii* and *P. aeruginosa*. According to clinical specimen type, a higher AMR was observed in *E. coli*, *K. pneumoniae*, and *P. aeruginosa* isolates from blood specimens. The most frequently detected carbapenemase-encoding gene in *E. coli* was *bla*_NDM_ (84%).

## 1. Introduction

In an effort to promote drug research and development, the World Health Organization (WHO) has identified and classified priority antibiotic-resistant pathogens into three groups on the basis of the urgency of the need for novel antibiotics. The first and the second groups (critical- and high-priority pathogens, respectively) include, among others, carbapenem-resistant and extended-spectrum beta-lactamase-producing *Klebsiella pneumoniae* and *Escherichia coli*, carbapenem-resistant *Acinetobacter baumannii* and *Pseudomonas aeruginosa*, vancomycin-resistant *Enterococcus faecium*, and methicillin-resistant *Staphylococcus aureus* [1]. The surveillance of antimicrobial resistance (AMR) in these critical- and high-priority pathogens requires a multidisciplinary approach, including national and local strategies [2]. Previous studies have reported that several factors, such as increased age, gender, and various demographics and comorbidities, increase the risk of infections due to antibiotic-resistant organisms [3,4].

In 2018, the Network for the Research and Surveillance of Drug Resistance (Red Temática de Investigación y Vigilancia de la Farmacorresistencia in Spanish; INVIFAR) was created to comprehensively study AMR in Mexico. Although previous studies have reported several aspects of antibiotic resistance in Mexico [5,6,7,8,9,10,11], including reports of an increase in drug resistance during the COVID-19 pandemic [12], there is limited information regarding AMR according to age and site of care at the time of infection.

In the present study, we assessed AMR in clinical isolates of *E. coli*, *K. pneumoniae*, *A. baumannii*, *P. aeruginosa*, *E. faecium*, and *S. aureus* from Mexico on the basis of clinical specimens from which the isolates were recovered, patient age and site of care, and the presence of carbapenemase-encoding genes in the included Gram negatives.

## 2. Materials and Methods

### 2.1. Participating Centers and Data Collection

A total of 43 centers across 18 Mexican states participated in this study. We collected data from 34 hospital center-based and 9 ambulatory-care microbiology laboratories. Data regarding each center’s total number of beds and intensive care unit (ICU) capacity, clinical specimens from which the studied isolates were recovered, and patient age and site of care were collected. AMR data from *E. coli*, *K. pneumoniae*, *A. baumannii*, *P. aeruginosa*, *E. faecium,* and *S. aureus* recovered from urine, respiratory, and blood specimens between 1 January and 31 March 2023 were included. Pathogens with the result of AMR data of more than 10 isolates were included.

Data from each laboratory were deposited into WHONET 2022^®^ software (WHO Collaborating Centre for the Surveillance of Antibiotic Resistance, Geneva, Switzerland). The extracted file was converted to the WHONET 2022 format through the data conversion utility BacLink 2022 (available online: http://www.whonet.org/, accessed on 1 June 2022). After conversion, all WHONET files for each hospital were combined and analyzed.

For the AMR analysis, only one isolate per patient was selected. The results were reported as antibiotic-susceptible, intermediate, or resistant according to the Clinical & Laboratory Standards Institute (CLSI) [13]. The data from patients were encrypted to protect personal information. Antimicrobial susceptibility testing (AST) was performed using amikacin (AMK), amoxicillin–clavulanic acid (AMC), ampicillin (AMP), aztreonam (ATM), ampicillin–sulbactam (SAM), cefepime (FEP), cefoxitin (FOX), ceftazidime (CAZ), ceftriaxone (CRO), cefuroxime (CXM), ciprofloxacin (CIP), clindamycin (CLI), cefotaxime (CTX), erythromycin (ERY), ertapenem (ETP), gentamicin (GEN), imipenem (IPM), linezolid (LNZ), levofloxacin (LVX), meropenem (MEM), oxacillin (OXA), tetracycline (TCY), tigecycline (TGC), piperacillin–tazobactam (TZP), tobramycin (TOB), trimethoprim–sulfamethoxazole (SXT), and vancomycin (VAN).

Using the WHONET software, Gram-negative isolates were classified as multi-drug-resistant (MDR) when non-susceptibility to at least one antibiotic in the three antimicrobial classes tested was documented, extensively drug-resistant (XDR) when non-susceptibility to at least one antibiotic in all but two or fewer antimicrobial classes was reported, and pan-drug-resistant (PDR) when non-susceptibility to all antibiotics in all antimicrobial classes tested was noted [14]. Because only one antimicrobial agent was evaluated for each antimicrobial class for some isolates, the categories of possible XDR and possible PDR were also described [14].

### 2.2. Site of Care, Age, and Clinical Specimens

The frequency of resistance to distinct antimicrobials was adjusted to the patient’s site of care (ICU, hospitalized medical/surgical non-ICU, emergency room, and outpatient setting) and age group (0–18 years, 19–59 years, and ≥60 years), and clinical specimens from which the studied isolates were cultured, such as respiratory (endotracheal aspirate and bronchial lavage), blood, and urine specimens. A comparison of antibiotic resistance between sites of care, age groups, and clinical specimens was performed using chi-square or Fisher’s exact test as appropriate. A two-tailed *p*-value ≤ 0.05 was considered statistically significant. The statistical analyses were performed using the MedCalc software, V 22.009.

### 2.3. Carbapenemase-Encoding Genes

As part of the active surveillance performed by the INVIFAR network, centers sent relevant carbapenem-resistant Gram negatives to the coordinating laboratory. In the clinical isolates received from centers, polymerase chain reaction (PCR) was performed to detect the most frequent carbapenemase-encoding genes previously reported in this population [15], that is, *bla*_NDM-1_, *bla*_KPC_, *bla*_VIM_, *bla*_IMP_, and *bla*_OXA-48-like_ [16,17] in *E. coli* and *K. pneumoniae* isolates; *bla*_OXA-23_ and *bla*_OXA-24_ in *A. baumannii* isolates [18,19]; and *bla*_VIM_, *bla*_IMP_, and *bla*_GES_ in *P. aeruginosa* isolates [10].

## 3. Results

### 3.1. Participating Centers and Study Population

During the study period, 43 centers reported data, of which 32/43 (74.4%) belong to the national public health care system and 11/43 (25.6%) belong to private practice health care system. To perform AST, 37/43 (83.7%) laboratories used commercial microdilution systems, of which 30 used the VITEK 2 system (Biomérieux, Marcy l’ Etoile, France), 5 used MicroScan WalkAway (Siemens Healthcare Diagnostics, West Sacramento, CA, USA), and 2 used the Phoenix System (Becton-Dickinson, Sparks, MD, USA). Six laboratories used the disk diffusion susceptibility method. The characteristics of the participating centers are listed in Table 1. Most clinical specimens (57%) were obtained from females. In total, 14% of patients belonged to the 0–18 years age group, whereas 49% and 37% belonged to the 19–59 years age group and ≥60 years age group, respectively. Most isolates were recovered from patients hospitalized in non-ICU settings (44%), followed by those in outpatient (32%), emergency room (16%), and ICU (8%) settings. A total of 6519 strains from all participating laboratories were analyzed: *E. coli (n* = 3985), *K. pneumoniae* (*n* = 775), *A. baumannii* (*n* = 163), *P. aeruginosa* (*n* = 781), *E. faecium* (*n* = 124), and *S. aureus* (*n* = 691).

### 3.2. Percentages of Resistance Detected in Critical- and High-Priority Pathogens/Phenotypes

**Figure 1:**
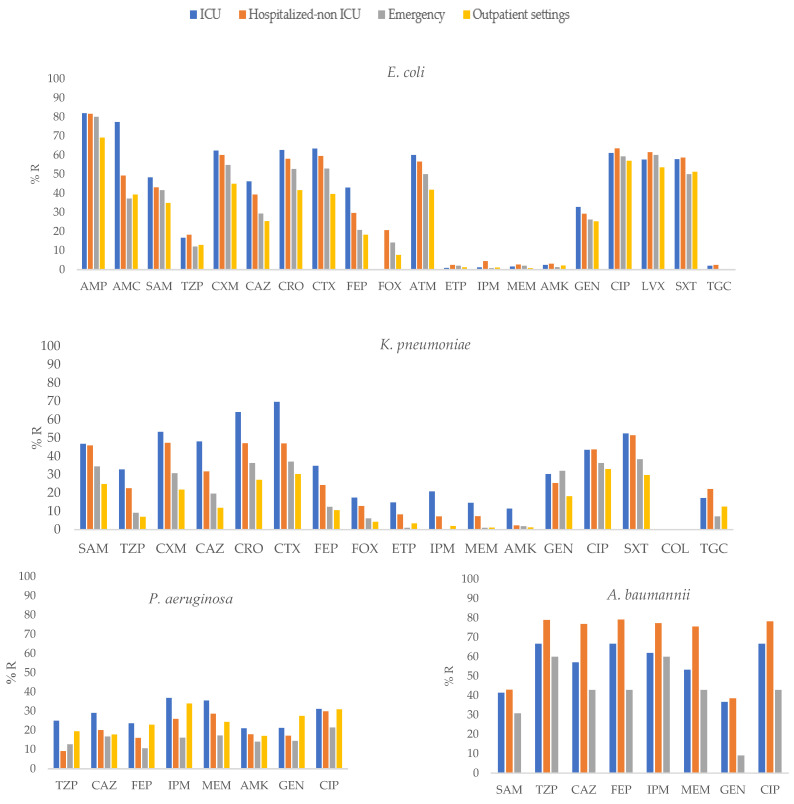
Percentages of antimicrobial resistance for selected Gram-negative pathogens at 43 centers according to sites of care. AMK: amikacin, AMC: amoxicillin–clavulanic acid, AMP: ampicillin, SAM: ampicillin–sulbactam, FEP: cefepime, FOX: cefoxitin, CAZ: ceftazidime, CRO: ceftriaxone, CXM: cefuroxime, CIP: ciprofloxacin, CTX: cefotaxime, ETP: ertapenem, GEN: gentamicin, IPM: imipenem, LVX: levofloxacin, MEM: meropenem, TGC: tigecycline, TZP: piperacillin–tazobactam, SXT: trimethoprim-sulfamethoxazole.

**Figure 2:**
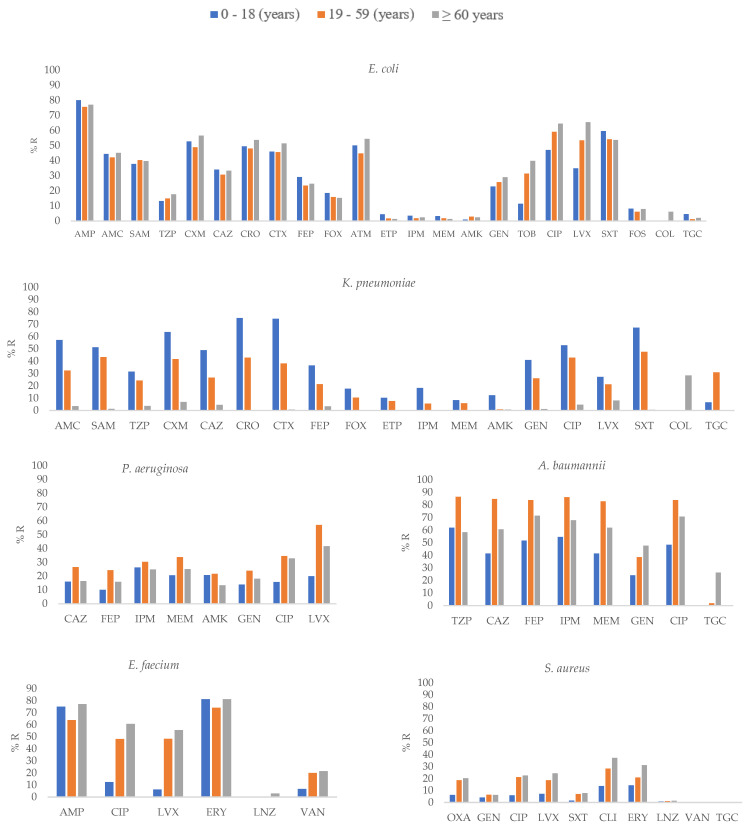
Percentages of antimicrobial resistance for selected pathogens at 43 centers according to age groups. AMK: amikacin, AMC: amoxicillin–clavulanic acid, AMP: ampicillin, SAM: ampicillin–sulbactam, FEP: cefepime, FOX: cefoxitin, CAZ: ceftazidime, CRO: ceftriaxone, CXM: cefuroxime, CIP: ciprofloxacin, CLI: Clindamycin, CTX: cefotaxime, ERY: erythromycin, ETP: ertapenem, GEN: gentamicin, IPM: imipenem, LNZ: linezolid, LVX: levofloxacin, MEM: meropenem, OXA: oxacillin, TGC: tigecycline, TZP: piperacillin–tazobactam, TOB: tobramycin, SXT: trimethoprim-sulfamethoxazole, VAN: vancomycin.

**Figure 3:**
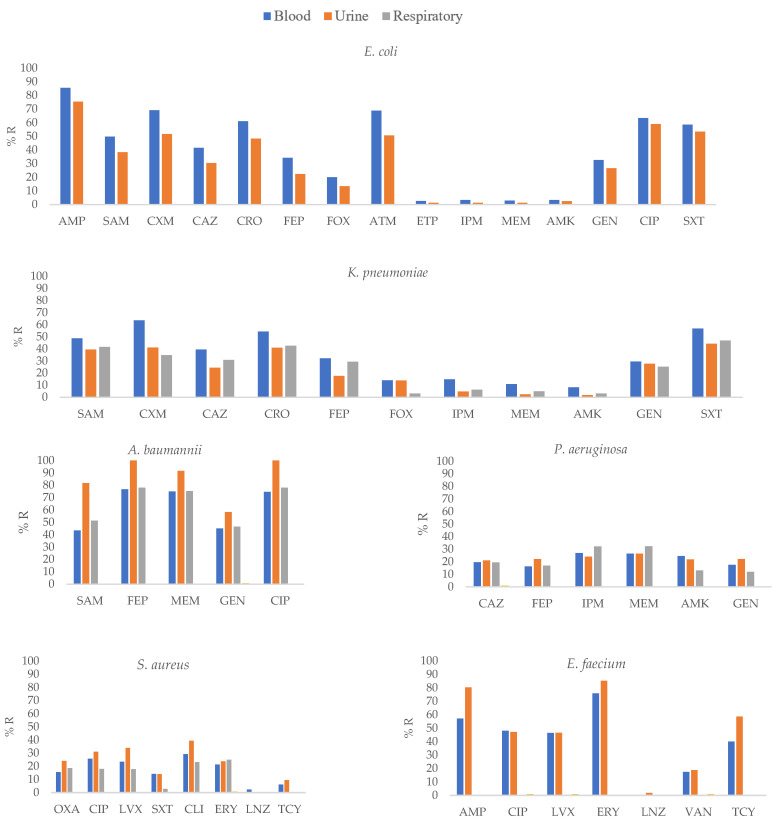
Percentages of antimicrobial resistance for selected Gram-negative pathogens at 43 centers according to clinical specimens. AMK: amikacin, AMC: amoxicillin–clavulanic acid, AMP: ampicillin, SAM: ampicillin–sulbactam, FEP: cefepime, FOX: cefoxitin, CAZ: ceftazidime, CRO: ceftriaxone, CXM: cefuroxime, CIP: ciprofloxacin, CLI: clindamycin, CTX: cefotaxime, ERY: erythromycin, ETP: ertapenem, GEN: gentamicin, IPM: imipenem, LNZ: linezolid, LVX: levofloxacin, MEM: meropenem, OXA: oxacillin, TGC: tigecycline, TZP: piperacillin–tazobactam, TOB: tobramycin, SXT: trimethoprim–sulfamethoxazole, VAN: vancomycin.

The results of the distribution of antibiotic resistance are shown in Supplementary Tables S1–S3 and Figure 1, Figure 2 and Figure 3. Third-generation cephalosporin resistance in *K. pneumoniae* was as high as 75% for CRO in isolates from the 0–18 years age group.

For *K. pneumoniae*, the highest frequency of carbapenem resistance was detected in 20.8% of the isolates recovered from ICU-admitted patients. For *A. baumannii*, carbapenem resistance was reported to be 86.2% in the 19–50 group and 36.8% for *P. aeruginosa* recovered from patients in the ICU. The methicillin resistance was found in 20.3% *S. aureus* isolates recovered from patients aged ≥60 years.

### 3.3. Antimicrobial Resistance of Selected Pathogens According to Site of Care

*E. coli* isolates obtained from ICU patients showed a higher frequency of resistance to AMP, AMC, SAM, CXM, CAZ, CRO, CTX, FEP, IPM, and GEN than other *E. coli* isolates (*p* ≤ 0.01; Supplementary Table S1, Figure 1).

In *K. pneumoniae*, isolates recovered from ICU patients showed higher resistance to SAM, TZP, CAZ, CRO, CTX, FEP, ETP, IPM, MEM, AMK, and SXT (*p* ≤ 0.01), than isolates from other groups.

In *P. aeruginosa*, isolates recovered from ICU patients showed higher resistance to TZP, FEP, IPM, and MEM (*p* ≤ 0.05) than isolates obtained from other groups. In contrast, in *A. baumannii*, isolates obtained from hospitalized non-ICU patients showed higher resistance to CAZ, CRO, MEM, FEP and CIP (*p* ≤ 0.01) than other isolates.

### 3.4. Antimicrobial Resistance of Selected Pathogens According to Patient Age Group

*E. coli* isolates recovered from the ≥60 years age group showed higher resistance to most antibiotics, including CXM, CIP, LVX (*p* ≤ 0.01) and TOB (*p* = 0.01; Supplementary Table S2, Figure 2). In contrast, in *K. pneumoniae*, isolates obtained from the 0–18 years age group showed higher resistance to SAM, CXM, CAZ, CRO, CTX, FEP, AMK, GEN, and SXT (*p* ≤ 0.01), ETP, and IPM (*p* ≤ 0.05). Regarding *A. baumannii*, higher resistance to CAZ, FEP, IPM, MEM, and CIP (*p* ≤ 0.01) in the 19-59 years group was found; similar results were observed for *P. aeruginosa*. For *E. faecium* and *S. aureus*, isolates recovered from the ≥60 years age group showed higher resistance to CIP and LVX (*p* ≤ 0.01) and OXA, CIP, LVX, CLI, and ERY (*p* ≤ 0.01), respectively.

### 3.5. Antimicrobial Resistance of Selected Pathogens According to Clinical Specimen Type

*E. coli* isolates from blood specimens showed higher resistance to SAM, CAZ, CRO, and FEP (*p* ≤ 0.01) and CXM, IPM, MEM, and GEN (*p* ≤ 0.05) than isolates from other clinical specimens. *K. pneumoniae* isolates from blood specimens showed higher resistance to CAZ, FEP, ETP, MEM, and AMK (*p* ≤ 0.01) and CXM, CRO, and IPM (*p* ≤ 0.05) than *K. pneumoniae* isolates from other clinical specimens (Supplementary Table S3, Figure 3).

In *P. aeruginosa*, isolates obtained from blood and urine specimens showed higher resistance to AMK and GEN (*p* ≤ 0.05) than other isolates. *S. aureus* isolates from blood specimens showed higher resistance to SXT (*p* ≤ 0.01) than isolates from other clinical specimens.

### 3.6. MDR, XDR, and PDR Isolates

MDR, true XDR, possible XDR, and possible PDR isolates were detected among samples obtained from patients in all studied settings. Isolates recovered from ICU patients showed the highest frequency of MDR in *E. coli* (62.9%), *K. pneumoniae* (50.5%), and *P. aeruginosa* (29.7%). For *A. baumannii*, the highest frequency of MDR was observed among isolates cultured from non-ICU hospitalized patients (79.1%; Table 2).

The highest frequency of possible XDR isolates of *E. coli* was detected in isolates from ambulatory-care patients (14%); for *K. pneumoniae* and *P. aeruginosa*, possible XDR was detected in 37.6% and 27.1% of the isolates cultured from ICU-admitted patients, respectively. The highest frequency of possible XDR isolates of *A. baumannii* was detected in isolates from hospitalized non-ICU patients (78.2%). True XDR *P. aeruginosa* was detected across samples obtained from patients in all settings (Table 2).

### 3.7. Carbapenemase-Encoding Genes

In *K. pneumoniae* and *E. coli*, the most frequently detected carbapenemase-encoding gene was *bla*_NDM_ (50%, 22/44 and 84%, 63/75, respectively) in the strains evaluated. The second most frequent carbapenemase-encoding genes detected in *K. pneumoniae* and *E. coli* were *bla*_KPC_ (27.3%, 12/44) and *bla*_OXA-48-like_ (12%, 9/75; Table 3), respectively. In *A. baumannii*, the most frequent carbapenemase-encoding gene was *bla*_OXA24_.

## 4. Discussion

The Infectious Diseases Society of America recognizes antimicrobial resistance as threat to human health worldwide [20]. In the present study, we studied AMR in five pathogenic species considered critical and high-priority by the WHO [1] via the analysis of consolidated data on antibiotic resistance by specimen, according to patient age and site of care, increasing the value and usefulness of the data generated.

Among the organisms/phenotypes to be surveyed according to the WHO recommendations, in *E. coli* and *K. pneumoniae*, the highest value of third-generation cephalosporin resistance was observed in isolates obtained from ICU patients (63.4%) and from the 0–18 years group (75%), respectively. A significant increase in the prevalence of extended-spectrum beta-lactamase-producing Enterobacterales in children has been reported in the USA, and this increase has been correlated with the spread of ST131 CTX-M-producing *E. coli* strains [21].

Recently, 24 *E. coli* strains from the same population were sequenced and the majority of them, 11 (45.8%), were detected to be ST2 (Pasteur)-ST167 (Warwick), followed by ST650 (Pasteur)-ST 361 (Warwick) (16.7%), with only one strain detected to be ST131, suggesting that ST131 has no impact in the alarmingly high levels of cephalosporin resistance.

In the present study, *K. pneumoniae* isolates obtained from ICU patients showed the highest carbapenem resistance (20.8%). This result is highly relevant because the hospital mortality of patients infected with carbapenem-resistant *K. pneumoniae* isolates has been reported to be 48%, in contrast with the 20% mortality reported for patients infected with carbapenem-susceptible *K. pneumoniae* [22].

AMR is considered one of the key determinants of patient outcome, and patients in the ICU are at a higher risk of acquiring antimicrobial-resistant infections, owing to the use of invasive devices, clinical condition, and increased exposure to antibiotics [23]. In our study, we detected higher antibiotic resistance in isolates obtained from patients in ICU settings than those in other settings for *E. coli* and *K. pneumoniae* (resistance to SAM, CAZ, CRO, CTX, FEP, and IPM; *p* ≤ 0.01) and for *P. aeruginosa* (resistance to TZP, FEP, IPM, and MEM, *p* ≤ 0.05).

The relevance of *E. coli* in the ICU has been reported previously. Although antibiotic resistance may affect any patient in the hospital, a nationwide study on bloodstream infections in ICUs in Swiss hospitals during 2008–2017 reported that the most common antibiotic-resistant species was *E. coli* (23.2%, 910), with resistance to first- and second-line antibiotics increasing linearly during hospitalization [24].

Previous studies have identified a predominance of *A. baumannii* in ICUs, especially in patients who have undergone invasive procedures and those with a prolonged ICU stay and prior use of broad-spectrum antimicrobial agents [25,26,27] constituting 7.9% of ventilator-associated pneumonia and up to 15.7% of bloodstream infections [28,29]. This bacterial species has been designated a human “red alarm” pathogen mainly because of its broad antibiotic resistance [30]. A high frequency of drug resistance in *A. baumannii* has been previously reported in Mexico [31,32,33,34,35,36,37], but there is little information on drug resistance in hospital settings. In our study, *A. baumannii* isolates from hospitalized non-ICU patients showed higher resistance to carbapenems and quinolones than isolates from ICU patients. It has been reported that the efficacy of antimicrobial agents is affected by hygiene quality [38]. The high drug resistance of this bacterial species in non-ICU hospitalized patients is relevant because it suggests that factors other than ICU stay such as antimicrobial stewardship, hand sanitization, and isolation of patients may play an important role in reducing infections by this bacterial species.

Some studies have investigated carbapenem resistance in Enterobacterales in Mexico, including carbapenemase production and genes encoding these enzymes, especially in *K. pneumoniae* and *E. coli* [7,39]. In the present study, we confirmed that, among Enterobacterales, *K. pneumoniae* show higher carbapenem resistance, and a high distribution of *bla*_NDM_ was observed in both *K. pneumoniae* and *E. coli*. The detection of *bla*_NDM_ in both *E. coli* and *K. pneumoniae* complicates the treatment of patients infected with these isolates because few therapeutic options are available for metallo-β-lactamase-producing bacteria; for example, the ceftazidime/avibactam + aztreonam combination [40] is not available in most centers in Mexico. A previous study that included a global collection of 81, 781 isolates of *Enterobacterales* collected from 39 countries in five geographic regions from 2012 to 2017 also reported that *K. pneumoniae* had a higher number of meropenem-nonsusceptible isolates (76.7%). In contrast, the majority of meropenem-nonsusceptible *Enterobacterales* were found to carry KPC-type carbapenemases (47.4%), followed by metallo-β-lactamases (20.6%) or OXA-48-like β-lactamases (19.0%) [41].

In a percentage of our isolates resistant to carbapenems, mainly Enterobacterales, we did not find any gene that codes for carbapenemase. Thus, carbapenem resistance may be porin- or efflux-pump-associated.

MDR bacteria are well-recognized to be one of the most important health problems. In a previous study on clinical samples in Iran, 16.50% of *P. aeruginosa* isolates and 74.75% of *A. baumannii* isolates were MDR [42]. Furthermore, a systematic review that included eight articles showed that the prevalence of MDR Gram-negative bacilli was from 11.2 to 59.1% in nursing home residents [43] and colonization by MDR *P. aeruginosa* and *A. baumannii* was reported in 5.4% and 15.0% of residents in long-term care facilities in North America [44]. In the present study, similar results were obtained, including clinical samples with 29.7% MDR isolates of *P. aeruginosa* and 79.1% MDR isolates of *A. baumannii*. MDR in bacterial species makes the control of infectious diseases difficult, worsening the effectiveness of treatment and increasing the likelihood of proliferation of resistant pathogens, leading to an extended time of infection in patients [45]. Thus, exhaustive infection control measures should be implemented when these organisms are detected.

Independent predictors of mortality have been associated with ICU stay, antimicrobial therapy, and clinical factors such as sepsis, the use of medical devices, and the presence of immunosuppression [46,47,48,49] that have also been associated with high multidrug resistance [50]. Our study did not analyze clinical data, but including these variables may provide a better idea of their impact on drug resistance in Mexican hospitals.

In our study, we performed AMR surveillance by collecting data from a limited number of sentinel healthcare facilities (43 centers), and the use of WHONET facilitated the analysis by obtaining the data directly from automated instruments. However, there are invisible areas in regions of the country that need to be included to have a better idea of the actual situation of drug resistance [51].

In our study, different AST methods were used in each laboratory. This variability introduced a bias in the results. However, to minimize the effect of this variation, all values were interpreted according to CLSI document M100-S33.

## 5. Conclusions

This study has certain limitations, including that colonizing and infection-causing isolates were not distinguished, the selection of the participating centers introduces possible biases, and the study includes data for a relatively short period (January to March 2023).

The highest carbapenem resistance in Enterobacterales and *P. aeruginosa* was detected in isolates from ICU patients, and the highest carbapenem resistance in *A. baumannii* was detected in isolates recovered from hospitalized non-ICU patients. Furthermore, the high cephalosporin resistance detected in the 0–18 years age group deserves special attention. The *bla*_NDM_ gene was the most frequently detected carbapenemase-encoding gene among carbapenem-resistant *K. pneumoniae* and *E. coli*, followed by *bla*_KPC_.

Our results may facilitate the implementation of direct and specific antibiotic resistance control measures according to the site of care and patient age group. They may have potential clinical implications by allowing the guidance of empirical therapies that may be more effective and more useful for the establishment of public health policies at a national level.

## Figures and Tables

**Table 1:** Distribution and characteristics of participating centers.

No.	State	Type	Level	Center Type	Total Beds	ICU Beds
1	Jalisco	Pu	Ter	Spe	760	45
2	Mexico City	Pu	Ter	Spe	245	15
3	Mexico State	Pu	Ter	Spe	238	28
4	Mexico City	Pu	Ter	Spe	234	22
5	Aguascalientes	Pu	Ter	Spe	218	23
6	Mexico State	Pu	Ter	Spe	210	8
7	Mexico City	Pu	Ter	Spe	193	15
8	Mexico City	Pu	Ter	Spe	146	8
9	Chiapas	Pu	Ter	Spe	90	15
10	Nuevo León	Pr	Ter	Spe	83	8
11	Sonora	Pu	Ter	Spe	60	6
12	Michoacán	Pr	Ter	Spe	55	11
13	Quintana Roo	Pr	Ter	Spe	54	4
14	Mexico State	Pu	Ter	Ped	180	8
15	Puebla	Pu	Ter	Ped	130	10
16	Coahuila	Pu	Sec	Ped	74	5
17	Sonora	Pu	Sec	Gen	290	12
18	Michoacán	Pu	Sec	Gen	250	24
19	Guanajuato	Pu	Sec	Gen	221	20
20	Sonora	Pu	Sec	Gen	171	4
21	Oaxaca	Pu	Sec	Gen	162	12
22	Mexico City	Pu	Sec	Gen	145	10
23	Chiapas	Pu	Sec	Gen	140	18
24	Sonora	Pu	Sec	Gen	122	4
25	Chiapas	Pu	Sec	Gen	120	25
26	Baja California Sur	Pu	Sec	Gen	120	17
27	Colima	Pu	Sec	Gen	105	4
28	Michoacán	Pu	Sec	Ped	100	7
29	Chihuahua	Pu	Sec	Gen	100	6
30	Jalisco	Pu	Sec	Gen	87	5
31	Sonora	Pu	Sec	Spe	35	4
32	Michoacán	Pr	Pri	Gen	32	3
33	Guerrero	Pu	Sec	Moth and Child	27	12
34	Quintana Roo	Pu	Sec	Moth and Child	20	20
35	Mexico State	Pr	Pri	ACL	0	0
36	Michoacán	Pr	Pri	ACL	0	0
37	Guerrero	Pr	Pri	ACL	0	0
38	Michoacán	Pr	Pri	ACL	0	0
39	Michoacán	Pr	Pri	ACL	0	0
40	Oaxaca	Pr	Pri	ACL	0	0
41	Nuevo León	Pu	Pri	ACL	0	0
42	Michoacán	Pr	Sec	ACL	0	0
43	Sonora	Pu	Pri	ACL	0	0

ICU: intensive care unit, Pu: public health care, Pr: private health care, Gen: general hospital, Spe: specialized center, Moth and Child: mother and child, Ped: pediatric center, ACL: ambulatory care laboratory.

**Table 2:** Distribution of MDR, possible XDR, and possible PDR bacteria among species studied.

ICU	MDR *n* (%)	Possible XDR *n* (%)	True XDR *n* (%)	Possible PDR *n* (%)	Total
*E. coli*	83 (62.9)	14 (10.6)	0 (0)	0 (0.0)	132
*K. pneumoniae*	47 (50.5)	35 (37.6)	0 (0)	2 (2.2)	93
*A. baumannii*	20 (66.7)	18 (60.0)	0 (0)	6 (20.0)	30
*P. aeruginosa*	35 (29.7)	32 (27.1)	2 (1.7)	15 (12.7)	118
Hospitalized non-ICU					
*E. coli*	775 (53.4)	199 (13.7)	0 (0)	0 (0.0)	1450
*K. pneumoniae*	152 (44.6)	106 (31.1)	0 (0)	2 (0.6)	341
*A. baumannii*	87 (79.1)	86 (78.2)	0 (0)	41 (37.3)	110
*P. aeruginosa*	87 (21.5)	79 (19.5)	5 (1.2)	46 (11.4)	405
Emergency					
*E. coli*	280 (48.6)	72 (12.5)	0 (0)	1 (0.2)	576
*K. pneumoniae*	40 (34.5)	25 (21.6)	0 (0)	0 (0.0)	116
*A. baumannii*	6 (42.9)	6 (42.9)	0 (0)	2 (14.3)	14
*P. aeruginosa*	19 (17.4)	14 (12.8)	1 (0.9)	4 (3.7)	109
Outpatients					
*E. coli*	744 (45.8)	228 (14.0	0 (0)	0 (0.0)	1625
*K. pneumoniae*	55 (28.1)	36 (18.4)	0 (0)	0 (0.0)	196
*P. aeruginosa*	30 (25.4)	2 (22.9)	1 (0.8)	7 (5.9)	118

MDR: multidrug-resistant, XDR: extensively drug-resistant, PDR: pan-drug-resistant.

**Table 3:** Distribution of genes encoding carbapenemase among bacterial species investigated in this study. ND—Not determined.

N	Species	*bla*_NDM_	*bla*_KPC_	*bla*_VIM_	*bla*_IMP_	*bla*_OXA48_	*bla*_GES_	*bla*_OXA23_	*bla*_OXA24_
22	*K. pneumoniae*	+	−	−	−	−	ND	ND	ND
12	*K. pneumoniae*	−	+	−	−	−	ND	ND	ND
2	*K. pneumoniae*	−	−	−	−	+	ND	ND	ND
8	*K. pneumoniae*	−	−	−	−	−	ND	ND	ND
57	*E. coli*	+	−	−	−	−	ND	ND	ND
6	*E. coli*	−	−	−	−	+	ND	ND	ND
3	*E. coli*	+	−	+	−	−	ND	ND	ND
3	*E. coli*	+	−	−	−	+	ND	ND	ND
1	*E. coli*	−	+	−	−	−	ND	ND	ND
1	*E. coli*	−	−	+	−	−	ND	ND	ND
4	*E. coli*	−	−	−	−	−	ND	ND	ND
47	*P. aeruginosa*	ND	ND	−	−	ND	+	ND	ND
32	*P. aeruginosa*	ND	ND	−	+	ND	−	ND	ND
11	*P. aeruginosa*	ND	ND	−	+	ND	+	ND	ND
11	*P. aeruginosa*	ND	ND	+	−	ND	−	ND	ND
1	*P. aeruginosa*	ND	ND	+	−	ND	+	ND	ND
1	*P. aeruginosa*	ND	ND	+	+	ND	−	ND	ND
100	*P. aeruginosa*	ND	ND	−	−	ND	−	ND	ND
47	*A. baumannii*	−	ND	ND	ND	ND	ND	−	+
32	*A. baumannii*	−	ND	ND	ND	ND	ND	+	−
5	*A. baumannii*	−	ND	ND	ND	ND	ND	+	+
1	*A. baumannii*	+	ND	ND	ND	ND	ND	+	−
10	*A. baumannii*	−	ND	ND	ND	ND	ND	−	−

## Data Availability

All research data is included in Supplementary Tables S1–S3.

## Supplementary Materials

The following supporting information can be downloaded at: https://www.mdpi.com/article/10.3390/pathogens12091144/s1, Table S1: Percentages of antimicrobial resistance for selected pathogens at 43 centers according to sites of care; Table S2: Percentages of antimicrobial resistance for selected pathogens at 43 centers according to age groups; Table S3: Percentages of antimicrobial resistance for selected Gram-negative pathogens at 43 centers according to clinical specimens.
