# Supplementary material for: Active Surveillance of Antimicrobial Resistance and Carbapenemase-Encoding Genes According to Sites of Care and Age Groups in Mexico: Results from the INVIFAR Network
Source: Pathogens. 2023 Sep 7;12(9):1144. doi: 10.3390/pathogens12091144 (PMC10537696; doi:10.3390/pathogens12091144)
Supplement: Supplementary file 1 [file pathogens-12-01144-s001.zip › pathogens-2566280-supplementary.pdf]

Supplementary Table S1. Percentages of antimicrobial resistance for selected pathogens at 43 centers according to sites of care.

		Intensive care unit				Hospitalized-non intensive care unit				Emergency room				Outpatient setting				
	**Antibiotic**	**n**	**%R**	**%I**	**%S**	**n**	**%R**	**%I**	**%S**	**n**	**%R**	**%I**	**%S**	**n**	**%R**	**%I**	**%S**	p
E. coli	AMP	72	81.9	1.4	16.7	883	81.7	0.7	17.7	381	80.1	1.0	18.9	977	69.2	2.0	28.8	≤0.01
	AMC	22	77.3	4.5	18.2	69	49.3	7.2	43.5	43	37.2	16.3	46.5	295	39.3	7.5	53.2	≤0.01
	SAM	91	48.4	11.0	40.7	1,248	43.1	5.2	51.7	483	41.6	4.3	54.0	987	34.9	2.6	62.5	≤0.01
	TZP	60	16.7	5.0	78.3	460	18.3	1.7	80.0	133	12.0	2.3	85.7	241	12.9	5.8	81.3	0.16
	CXM	69	62.3	1.4	36.2	803	60.0	2.1	37.9	343	54.8	2.9	42.3	1,037	44.9	9.5	45.6	≤0.01
	CAZ	106	46.2	0.0	53.8	1,308	39.3	1.4	59.3	525	29.3	2.5	68.2	1,240	25.4	2.9	71.7	≤0.01
	CRO	126	62.7	0.8	36.5	1,355	58.0	0.1	41.9	560	52.7	0.0	47.3	1,496	41.6	0.2	58.2	≤0.01
	CTX	41	63.4	0.0	36.6	744	59.5	0.3	40.2	327	52.9	0.6	46.5	947	39.6	0.3	60.1	≤0.01
	FEP	128	43.0	6.3	50.8	1,368	29.6	6.4	64.0	559	20.8	5.9	73.3	1,282	18.3	6.6	75.2	≤0.01
	FOX	20	0.0	15.0	85.0	286	20.6	5.6	73.8	113	14.2	4.4	81.4	234	7.7	1.7	90.6	≤0.01
	ATM	30	60.0	0.0	40.0	76	56.6	0.0	43.4	32	50.0	0.0	50.0	98	41.8	0.0	58.2	0.16
	ETP	125	0.8	0.8	98.4	1,353	2.4	0.1	97.5	559	2.0	0.0	98.0	1,465	1.2	0.1	98.7	0.11
	IPM	83	1.2	0.0	98.8	456	4.4	0.2	95.4	131	0.8	0.0	99.2	462	1.1	0.0	98.9	≤0.01
	MEM	128	1.6	0.0	98.4	1,371	2.6	0.0	97.4	560	2.0	0.0	98.0	1,527	0.7	0.0	99.3	≤0.01
	AMK	124	2.4	4.0	93.5	1,355	3.0	1.6	95.4	552	1.3	1.4	97.3	1,434	2.1	2.7	95.2	0.11
	GEN	113	32.7	0.0	67.3	1,328	29.2	0.8	70.0	549	26.2	0.4	73.4	1,506	25.3	1.0	73.7	0.05
	CIP	126	61.1	10.3	28.6	892	63.5	6.4	30.2	560	59.3	8.4	32.3	1,556	57.0	7.3	35.7	0.02
	LVX	33	57.6	15.2	27.3	130	61.5	6.2	32.3	60	60.0	5.0	35.0	400	53.5	2.8	43.8	0.399
	SXT	83	57.8	0.0	42.2	979	58.6	0.0	41.4	314	50.0	0.0	50.0	1,337	51.2	0.1	48.6	≤0.01
K. pneumoniae	SAM	77	46.8	3.9	49.4	281	45.9	0.7	53.4	93	34.4	3.2	62.4	129	24.8	0.8	74.4	≤0.01
	TZP	58	32.8	10.3	56.9	142	22.5	11.3	66.2	44	9.1	13.6	77.3	29	6.9	3.4	89.7	≤0.01
	CXM	30	53.3	3.3	43.3	150	47.3	2.7	50.0	52	30.8	3.8	65.4	23	21.7	0.0	78.3	0.02
	CAZ	77	48.1	2.6	49.4	309	31.7	7.1	61.2	97	19.6	7.2	73.2	152	11.8	3.9	84.2	≤0.01
	CRO	89	64.0	0.0	36.0	329	47.1	0.0	52.9	113	36.3	0.0	63.7	177	27.1	1.1	71.8	≤0.01
	CTX	23	69.6	0.0	30.4	149	47.0	0.7	52.3	54	37.0	0.0	63.0	119	30.3	0.8	68.9	≤0.01
	FEP	89	34.8	5.6	59.6	330	24.2	3.3	72.4	113	12.4	2.7	85.0	161	10.6	1.9	87.6	≤0.01
	FOX	23	17.4	0.0	82.6	78	12.8	2.6	84.6	33	6.1	3.0	90.9	24	4.2	0.0	95.8	0.356
	ETP	88	14.8	0.0	85.2	329	8.2	0.3	91.5	113	0.9	0.0	99.1	179	3.4	0.0	96.6	≤0.01
	IPM	53	20.8	0.0	79.2	139	7.2	0.0	92.8	38	0.0	0.0	100.0	52	1.9	1.9	96.2	≤0.01
	MEM	89	14.6	1.1	84.3	330	7.3	0.0	92.7	113	0.9	0.0	99.1	186	1.1	0.0	98.9	≤0.01
	AMK	88	11.4	2.3	86.4	320	2.2	0.6	97.2	112	1.8	0.9	97.3	168	1.2	1.2	97.6	≤0.01
	GEN	89	30.3	1.1	68.5	315	25.4	0.3	74.3	103	32.0	0.0	68.0	171	18.1	1.8	80.1	0.04
	CIP	85	43.5	5.9	50.6	270	43.7	6.3	50.0	113	36.3	1.8	61.9	188	33.0	6.9	60.1	0.09
	SXT	40	52.5	0.0	47.5	206	51.5	0.0	48.5	60	38.3	0.0	61.7	151	29.8	0.7	69.5	≤0.01
P. aeruginosa	TZP	68	25.0	10.3	64.7	206	9.2	10.2	80.6	55	12.7	10.9	76.4	41	19.5	2.4	78.0	0.01
	CAZ	100	29.0	8.0	63.0	374	20.1	4.3	75.7	102	16.7	3.9	79.4	90	17.8	3.3	78.9	0.12
	FEP	110	23.6	7.3	69.1	381	16.0	6.0	78.0	104	10.6	3.8	85.6	109	22.9	5.5	71.6	0.03
	IPM	76	36.8	0.0	63.2	216	25.9	0.9	73.1	56	16.1	3.6	80.4	56	33.9	3.6	62.5	0.04
	MEM	110	35.5	5.5	59.1	381	28.6	7.3	64.0	104	17.3	5.8	76.9	111	24.3	2.7	73.0	0.02
	AMK	105	21.0	1.9	77.1	368	17.9	0.8	81.3	100	14.0	1.0	85.0	105	17.1	1.9	81.0	0.63
	GEN	85	21.2	9.4	69.4	331	17.2	7.3	75.5	83	14.5	4.8	80.7	80	27.5	5.0	67.5	0.12
	CIP	106	31.1	0.0	68.9	308	29.9	3.6	66.6	103	21.4	1.0	77.7	110	30.9	2.7	66.4	0.33
	LVX	13	30.8	0.0	69.2	24	29.2	0.0	70.8	ND	ND	ND	ND	24	50.0	4.2	45.8	0.28
A. baumannii	SAM	29	41.4	17.2	41.4	107	43.0	33.6	23.4	13	30.8	7.7	61.5	NO	NO	NO	NO	0.70
	TZP	21	66.7	4.8	28.6	76	78.9	0.0	21.1	10	60.0	0.0	40.0	NO	NO	NO	NO	0.27
	CAZ	28	57.1	3.6	39.3	108	76.9	5.6	17.6	14	42.9	14.3	42.9	NO	NO	NO	NO	≤0.01

	FEP	30	66.7	0.0	33.3	110	79.1	0.9	20.0	14	42.9	0.0	57.1	NO	NO	NO	NO	0.01
	IPM	21	61.9	0.0	38.1	75	77.3	0.0	22.7	10	60.0	0.0	40.0	NO	NO	NO	NO	0.24
	MEM	30	53.3	3.3	43.3	110	75.5	0.9	23.6	14	42.9	0.0	57.1	NO	NO	NO	NO	≤0.01
	GEN	30	36.7	6.7	56.7	109	38.5	17.4	44.0	11	9.1	9.1	81.8	NO	NO	NO	NO	0.15
	CIP	30	66.7	0.0	33.3	110	78.2	0.0	21.8	14	42.9	0.0	57.1	NO	NO	NO	NO	0.01
S. aureus	OXA	95	17.9	0.0	82.1	262	18.3	0.0	81.7	122	19.7	0.0	80.3	143	10.5	0.0	89.5	0.15
	FOX	15	6.7	0.0	93.3	31	12.9	0.0	87.1	18	5.6	0.0	94.4	ND	ND	ND	ND	0.64
	GEN	87	3.4	1.1	95.4	245	6.1	5.3	88.6	97	7.2	5.2	87.6	90	5.6	2.2	92.2	0.73
	CIP	88	12.5	1.1	86.4	221	20.4	1.8	77.8	97	22.7	4.1	73.2	106	10.4	0.0	89.6	0.04
	LVX	71	12.7	0.0	87.3	254	20.1	0.4	79.5	115	20.0	0.0	80.0	122	10.7	0.8	88.5	0.08
	SXT	92	3.3	0.0	96.7	253	7.1	0.0	92.9	113	3.5	0.0	96.5	146	5.5	0.0	94.5	0.39
	CLI	90	20.0	0.0	80.0	246	28.9	0.0	71.1	109	28.4	0.0	71.6	120	25.0	0.0	75.0	0.39
	ERY	94	19.1	0.0	80.9	257	24.1	0.8	75.1	119	23.5	0.0	76.5	133	16.5	0.0	83.5	0.31
	LNZ	96	0.0	0.0	100.0	263	2.3	0.0	97.7	122	0.8	0.0	99.2	144	0.7	0.0	99.3	0.27
	VAN	94	1.1	1.1	97.9	252	2.4	1.6	96.0	115	1.7	0.9	97.4	124	0.0	3.2	96.8	0.35
E. faecium	AMP	ND	ND	ND	ND	69	71.0	0.0	29.0	18	77.8	0.0	22.2	13	53.8	0.0	46.2	0.34
	CIP	ND	ND	ND	ND	59	54.2	15.3	30.5	18	44.4	27.8	27.8	15	26.7	46.7	26.7	0.15
	LVX	ND	ND	ND	ND	69	53.6	4.3	42.0	18	38.9	22.2	38.9	16	25.0	0.0	75.0	0.09
	ERY	ND	ND	ND	ND	67	79.1	3.0	17.9	17	82.4	5.9	11.8	14	64.3	0.0	35.7	0.42
	LNZ	ND	ND	ND	ND	69	0.0	0.0	100.0	18	0.0	5.6	94.4	14	7.1	0.0	92.9	0.04
	VAN	ND	ND	ND	ND	58	24.1	0.0	75.9	16	6.3	0.0	93.8	11	18.2	0.0	81.8	0.28

AMK: amikacin, AMC: amoxicillin-clavulanic acid, AMP: ampicillin, SAM: ampicillin-sulbactam, FEP: cefepime, FOX: cefoxitin, CAZ: ceftazidime, CRO: ceftriaxone, CXM: cefuroxime, CIP: ciprofloxacin, CLI: Clindamycin, CTX: cefotaxime, ERY: erythromycin, ETP: ertapenem, GEN: gentamicin, IPM: imipenem, LNZ: linezolid, LVX: levofloxacin, MEM: meropenem, OXA: oxacillin, TGC: tigecycline, TZP: piperacillin-tazobactam, TOB: tobramycin, and SXT: trimethoprim-sulfamethoxazole, VAN: vancomycin. ND: No data. Comparison of antibiotic resistance between sites of care was performed using chi-square, or Fisher's exact test as appropriate. A two-tailed *p*-value <0.05 was considered statistically significant.

Supplementary Table S2. Percentages of antimicrobial resistance for selected pathogens at 43 centers according to age groups.

		0-18 (years)				19-59 (years)				≥60 years				p
	**Antibiotic**	**n**	**%R**	**%I**	**%S**	**n**	**%R**	**%I**	**%S**	**n**	**%R**	**%I**	**%S**	
E. coli	AMP	245	80.0	1.6	18.4	1,108	75.6	1.4	22.9	1,091	77.1	1.6	21.3	0.31
	AMC	72	44.4	2.8	52.8	178	42.1	10.1	47.8	173	45.1	11.0	43.9	0.85
	SAM	254	37.8	8.3	53.9	1,410	40.3	4.2	55.5	1,364	39.7	3.0	57.3	0.75
	TZP	106	13.2	5.7	81.1	402	14.9	1.7	83.3	368	17.7	2.7	79.6	0.42
	CXM	203	52.7	5.4	41.9	1,039	48.8	5.8	45.4	1,111	56.5	5.7	37.8	≤0.01
	CAZ	306	34.0	0.3	65.7	1,512	30.6	1.9	67.5	1,455	33.3	2.3	64.3	0.22
	CRO	346	49.4	0.3	50.3	1,642	48.1	0.1	51.8	1,656	53.6	0.2	46.2	0.01

	CTX	211	46.0	0.0	54.0	1,010	45.6	0.3	54.1	1,000	51.4	0.4	48.2	0.03
	FEP	323	29.1	3.4	67.5	1,582	23.4	5.9	70.7	1,540	24.7	7.1	68.2	0.09
	FOX	65	18.5	7.7	73.8	259	15.8	2.7	81.5	256	15.2	4.3	80.5	0.82
	ATM	30	50.0	0.0	50.0	105	44.8	0.0	55.2	136	54.4	0.0	45.6	0.33
	ETP	343	4.4	0.3	95.3	1,631	1.6	0.2	98.2	1,630	1.3	0.1	98.6	≤0.01
	IPM	117	3.4	0.9	95.7	460	1.7	0.0	98.3	507	2.4	0.0	97.6	0.52
	MEM	347	3.2	0.0	96.8	1,660	1.7	0.0	98.3	1,682	1.3	0.0	98.7	0.05
	AMK	343	0.9	3.2	95.9	1,613	2.9	1.9	95.2	1,617	2.4	2.1	95.5	0.08
	GEN	341	22.9	0.3	76.8	1,616	25.7	0.8	73.5	1,641	29.0	0.9	70.1	0.02
	TOB	35	11.4	5.7	82.9	118	31.4	6.8	61.9	113	39.8	5.3	54.9	0.01
	CIP	355	47.0	7.6	45.4	1,431	59.0	6.3	34.7	1,471	64.4	6.7	28.8	≤0.01
	LVX	66	34.8	4.5	60.6	236	53.4	5.5	41.1	298	65.4	3.7	30.9	≤0.01
	SXT	277	59.6	0.4	40.1	1,205	54.2	0.0	45.8	1,258	53.7	0.1	46.3	0.20
K. pneumoniae	AMC	14	57.1	7.1	35.7	34	32.4	8.8	58.8	28	28.6	3.6	67.9	0.17
	SAM	84	51.2	3.6	45.2	305	43.3	1.0	55.7	223	32.7	1.3	65.9	≤0.01
	TZP	60	31.7	16.7	51.7	131	24.4	12.2	63.4	82	14.6	3.7	81.7	0.052
	CXM	44	63.6	4.5	31.8	168	41.7	1.2	57.1	158	36.1	7.0	57.0	0.01
	CAZ	88	48.9	8.0	43.2	322	26.7	6.5	66.8	239	20.9	4.6	74.5	≤0.01
	CRO	108	75.0	0.0	25.0	348	42.8	0.3	56.9	277	33.2	0.4	66.4	≤0.01
	CTX	43	74.4	0.0	25.6	173	38.2	0.6	61.3	153	36.6	0.7	62.7	≤0.01
	FEP	107	36.4	5.6	57.9	345	21.4	4.3	74.2	266	13.5	3.4	83.1	≤0.01
	FOX	17	17.6	5.9	76.5	76	10.5	2.6	86.8	53	11.3	0.0	88.7	0.70
	ETP	107	10.3	0.0	89.7	351	7.7	0.3	92.0	275	3.6	0.0	96.4	0.03
	IPM	49	18.4	0.0	81.6	126	5.6	0.8	93.7	98	6.1	0.0	93.9	0.01
	MEM	108	8.3	0.9	90.7	353	5.9	0.3	93.8	281	3.2	0.0	96.8	0.09
	AMK	105	12.4	0.0	87.6	342	0.9	1.5	97.7	265	1.5	0.8	97.7	≤0.01
	GEN	105	41.0	0.0	59.0	336	26.2	0.6	73.2	260	20.8	1.2	78.1	≤0.01
	CIP	106	52.8	6.6	40.6	317	42.9	5.7	51.4	256	34.8	4.7	60.5	0.01
	LVX	11	27.3	0.0	72.7	33	21.2	9.1	69.7	37	21.6	8.1	70.3	0.91
	SXT	61	67.2	0.0	32.8	223	47.5	0.0	52.5	190	33.7	0.5	65.8	≤0.01
A. baumannii	TZP	21	61.9	4.8	33.3	59	86.4	0.0	13.6	24	58.3	0.0	41.7	≤0.01
	CAZ	29	41.4	13.8	44.8	85	84.7	1.2	14.1	33	60.6	12.1	27.3	≤0.01
	FEP	29	51.7	3.4	44.8	87	83.9	0.0	16.1	42	71.4	0.0	28.6	≤0.01
	IPM	22	54.5	0.0	45.5	58	86.2	0.0	13.8	28	67.9	0.0	32.1	≤0.01
	MEM	29	41.4	0.0	58.6	87	82.8	0.0	17.2	42	61.9	4.8	33.3	≤0.01
	AMK	ND	ND	ND	ND	15	6.7	0.0	93.3	12	50.0	8.3	41.7	0.01

	GEN	29	24.1	0.0	75.9	83	38.6	21.7	39.8	42	47.6	9.5	42.9	0.13
	CIP	29	48.3	0.0	51.7	87	83.9	0.0	16.1	41	70.7	0.0	29.3	≤0.01
	SXT	ND	ND	ND	ND	28	78.6	0.0	21.4	18	83.3	0.0	16.7	0.69
P aeruginosa	TZP	85	15.3	12.9	71.8	165	14.5	12.7	72.7	115	11.3	5.2	83.5	0.66
	CAZ	150	16.0	8.0	76.0	287	26.5	3.1	70.4	226	16.4	4.4	79.2	≤0.01
	FEP	159	10.1	5.0	84.9	304	24.3	7.2	68.4	247	15.8	4.5	79.8	≤0.01
	IPM	95	26.3	3.2	70.5	168	30.4	0.6	69.0	129	24.8	0.0	75.2	0.54
	MEM	160	20.6	7.5	71.9	306	33.7	6.5	59.8	247	25.1	4.5	70.4	≤0.01
	AMK	150	20.7	1.3	78.0	296	21.6	1.0	77.4	239	13.4	1.3	85.4	0.04
	GEN	151	13.9	7.3	78.8	234	23.9	8.1	67.9	199	18.1	4.5	77.4	0.04
	CIP	159	15.7	1.3	83.0	258	34.5	2.3	63.2	216	32.9	3.7	63.4	≤0.01
	LVX	20	20.0	0.0	80.0	14	57.1	0.0	42.9	24	41.7	4.2	54.2	0.08
S. aureus	OXA	125	6.4	0.0	93.6	347	18.7	0.0	81.3	138	20.3	0.0	79.7	≤0.01
	GEN	120	4.2	3.3	92.5	278	6.5	4.3	89.2	111	6.3	6.3	87.4	0.65
	CIP	115	6.1	2.6	91.3	272	21.3	1.5	77.2	115	22.6	0.9	76.5	≤0.01
	LVX	110	7.3	0.0	92.7	322	18.6	0.6	80.7	131	24.4	0.0	75.6	≤0.01
	SXT	125	1.6	0.0	98.4	336	7.1	0.0	92.9	127	7.9	0.0	92.1	0.06
	CLI	123	13.8	0.0	86.2	320	28.4	0.0	71.6	110	37.3	0.0	62.7	≤0.01
	ERY	125	14.4	0.0	85.6	340	20.9	0.6	78.5	125	31.2	0.0	68.8	≤0.01
	LNZ	125	0.8	0.0	99.2	345	1.2	0.0	98.8	138	1.4	0.0	98.6	0.89
	VAN	122	0.0	0.0	100	320	0.0	0.0	100	131	0.0	0.0	100	NA
E, faecium	AMP	16	75.0	0.0	25.0	58	63.8	0.0	36.2	35	77.1	0.0	22.9	0.35
	CIP	16	12.5	62.5	25.0	52	48.1	23.1	28.8	33	60.6	9.1	30.3	≤0.01
	LVX	16	6.3	18.8	75.0	60	48.3	5.0	46.7	36	55.6	5.6	38.9	≤0.01
	ERY	16	81.3	6.3	12.5	58	74.1	1.7	24.1	32	81.3	3.1	15.6	0.68
	LNZ	16	0.0	0.0	100.0	59	0.0	1.7	98.3	35	2.9	0.0	97.1	0.34
	VAN	15	6.7	0.0	93.3	50	20.0	0.0	80.0	28	21.4	0.0	78.6	0.44

AMK: amikacin, AMC: amoxicillin-clavulanic acid, AMP: ampicillin, ATM: aztreonam; SAM: ampicillin-sulbactam, FEP: cefepime, FOX: cefoxitin, CAZ: ceftazidime, COL: colistin; CRO: ceftriaxone, CXM: cefuroxime, CIP: ciprofloxacin, CLI: Clindamycin, CTX: cefotaxime, ERY: erythromycin, ETP: ertapenem, GEN: gentamicin, IPM: imipenem, LNZ: linezolid, LVX: levofloxacin, MEM: meropenem, OXA: oxacillin, TGC: tigecycline, TZP: piperacillin-tazobactam, TOB: tobramycin, and SXT: trimethoprim-sulfamethoxazole, VAN: vancomycin. Comparison of antibiotic resistance between age groups was performed using chi-square, or Fisher's exact test as appropriate. A two-tailed *p*-value <0.05 was considered statistically significant.

Supplementary Table S3. Percentages of antimicrobial resistance for selected Gram-negative pathogens at 43 centers according to clinical specimens.

		Blood				Urine				Respiratory				
	**Antibiotic**	**n**	**%R**	**%I**	**%S**	**n**	**%R**	**%I**	**%S**	**n**	**%R**	**%I**	**%S**	p
E. coli	AMP	69	85.5	0.0	14.5	2,202	75.4	1.7	22.9	ND	ND	ND	ND	0.05
	SAM	241	49.8	5.4	44.8	2,459	38.3	4.1	57.5	ND	ND	ND	ND	≤0.01
	CXM	55	69.1	5.5	25.5	2,172	51.6	5.9	42.5	ND	ND	ND	ND	0.01
	CAZ	253	41.5	1.2	57.3	2,685	30.4	2.1	67.5	ND	ND	ND	ND	≤0.01
	CRO	277	61.0	0.0	39.0	3,048	48.3	0.2	51.5	ND	ND	ND	ND	≤0.01
	FEP	275	34.2	6.9	58.9	2,785	22.3	6.2	71.5	ND	ND	ND	ND	≤0.01
	FOX	140	20.0	4.3	75.7	420	13.3	2.9	83.8	ND	ND	ND	ND	0.06
	ATM	16	68.8	6.3	25.0	172	50.6	0.0	49.4	ND	ND	ND	ND	0.16
	ETP	273	2.6	0.0	97.4	3,001	1.2	0.1	98.7	ND	ND	ND	ND	0.06
	IPM	209	3.3	0.0	96.7	772	1.2	0.0	98.8	ND	ND	ND	ND	0.03
	MEM	276	2.9	0.0	97.1	3,026	1.2	0.0	98.8	ND	ND	ND	ND	0.02
	AMK	272	3.3	4.0	92.6	2,975	2.4	2.1	95.5	ND	ND	ND	ND	0.35
	GEN	270	32.6	1.1	66.3	3,002	26.6	0.8	72.7	ND	ND	ND	ND	0.03
	CIP	216	63.4	6.5	30.1	2,653	59.1	7.4	33.6	ND	ND	ND	ND	0.21
	SXT	82	58.5	0.0	41.5	2,342	53.5	0.1	46.5	ND	ND	ND	ND	0.37
K. pneumoniae	AMC	ND	ND	ND	ND	37	32.4	10.8	56.8	19	42.1	0.0	57.9	0.47
	SAM	129	48.8	2.3	48.8	310	37.1	1.9	61.0	77	41.6	0.0	58.4	0.07
	TZP	102	26.5	16.7	56.9	73	15.1	11.0	74.0	72	25.0	2.8	72.2	0.18
	CXM	33	63.6	3.0	33.3	270	40.4	4.4	55.2	23	34.8	0.0	65.2	0.03
	CAZ	127	39.4	3.9	56.7	344	23.5	6.4	70.1	78	30.8	5.1	64.1	≤0.01
	CRO	147	54.4	0.0	45.6	381	40.2	0.5	59.3	101	42.6	0.0	57.4	0.01
	CTX	31	67.7	0.0	32.3	253	38.7	0.8	60.5	17	41.2	5.9	52.9	0.01
	FEP	146	32.2	4.1	63.7	358	16.2	4.7	79.1	102	29.4	2.9	67.6	≤0.01
	FOX	43	14.0	2.3	83.7	58	13.8	3.4	82.8	32	3.1	0.0	96.9	0.25
	ETP	147	12.9	0.0	87.1	378	2.9	0.3	96.8	102	8.8	1.0	90.2	≤0.01
	IPM	88	14.8	0.0	85.2	84	4.8	0.0	95.2	80	6.3	0.0	93.8	0.04

	MEM	147	10.9	0.7	88.4	380	2.4	0.3	97.4	102	4.9	0.0	95.1	≤0.01
	AMK	144	8.3	0.7	91.0	363	1.7	0.8	97.5	97	3.1	1.0	95.9	≤0.01
	GEN	146	29.5	0.7	69.9	371	26.7	1.1	72.2	83	25.3	0.0	74.7	0.75
	CIP	135	43.0	5.2	51.9	341	41.3	5.6	53.1	95	38.9	3.2	57.9	0.83
	SXT	58	56.9	0.0	43.1	271	42.1	0.4	57.6	49	46.9	0.0	53.1	0.12
A. baumannii	SAM	53	43.4	34.0	22.6	11	81.8	9.1	9.1	70	51.4	21.4	27.1	0.07
	TZP	44	81.8	2.3	15.9	ND	ND	ND	ND	56	78.6	0.0	21.4	0.68
	CAZ	ND	ND	ND	ND	11	100	0.0	0.0	68	75.0	2.9	22.1	0.06
	FEP	60	76.7	0.0	23.3	12	100	0.0	0.0	73	78.1	1.4	20.5	0.18
	IPM	47	80.9	0.0	19.1	ND	ND	ND	ND	59	79.7	0.0	20.3	0.88
	MEM	60	75.0	0.0	25.0	12	91.7	0.0	8.3	73	75.3	1.4	23.3	0.43
	GEN	60	45.0	8.3	46.7	12	58.3	8.3	33.3	71	46.5	11.3	42.3	0.70
	CIP	59	74.6	0.0	25.4	12	100	0.0	0.0	73	78.1	0.0	21.9	0.15
P. aeruginosa	TZP	72	11.1	11.1	77.8	ND	ND	ND	ND	153	18.3	9.2	72.5	0.17
	CAZ	97	19.6	3.1	77.3	223	21.1	4.5	74.4	200	19.5	7.0	73.5	0.91
	FEP	105	16.2	4.8	79.0	244	22.1	4.5	73.4	213	16.9	3.8	79.3	0.26
	IPM	74	27.0	0.0	73.0	96	24.0	0.0	76.0	168	32.1	3.6	64.3	0.35
	MEM	106	26.4	3.8	69.8	246	26.4	4.1	69.5	213	32.4	6.1	61.5	0.314
	AMK	102	24.5	0.0	75.5	242	21.9	1.7	76.4	200	13.0	1.0	86.0	0.02
	GEN	97	17.5	6.2	76.3	226	22.1	6.2	71.7	151	11.9	9.3	78.8	0.04
	CIP	95	25.3	0.0	74.7	184	35.3	2.2	62.5	ND	ND	ND	ND	0.09
S. aureus	OXA	128	15.6	0.0	84.4	ND	ND	ND	ND	177	18.6	0.0	81.4	0.43
	CIP	109	25.7	2.8	71.6	ND	ND	ND	ND	134	17.9	2.2	79.9	0.14
	LVX	119	23.5	0.0	76.5	ND	ND	ND	ND	157	17.8	0.6	81.5	0.24
	SXT	120	14.2	0.0	85.8	ND	ND	ND	ND	180	2.8	0.0	97.2	≤0.01
	CLI	123	29.3	0.0	70.7	ND	ND	ND	ND	173	23.1	0.0	76.9	0.23
	ERY	127	21.3	0.0	78.7	ND	ND	ND	ND	180	25.0	0.6	74.4	0.44
	LNZ	128	2.3	0.0	97.7	ND	ND	ND	ND	182	0.5	0.0	99.5	0.24
	VAN	126	0.0	2.4	97.6	ND	ND	ND	ND	177	0.0	0.0	100.0	NA
	TCY	49	6.1	0.0	93.9	ND	ND	ND	ND	52	0.0	0.0	100.0	0.12

E. faecium	AMP	28	57.1	0.0	42.9	56	80.4	0.0	19.6	ND	ND	ND	ND	0.02
	CIP	25	48.0	12.0	40.0	51	47.1	31.4	21.6	ND	ND	ND	ND	0.94
	LVX	28	46.4	0.0	53.6	58	46.6	6.9	46.6	ND	ND	ND	ND	0.99
	ERY	29	75.9	6.9	17.2	54	85.2	1.9	13.0	ND	ND	ND	ND	0.29
	LNZ	29	0.0	0.0	100.0	56	1.8	1.8	96.4	ND	ND	ND	ND	0.47
	VAN	23	17.4	0.0	82.6	48	18.8	0.0	81.3	ND	ND	ND	ND	0.89
	TCY	10	40.0	0.0	60.0	29	58.6	0.0	41.4	ND	ND	ND	ND	0.31

AMK: amikacin, AMC: amoxicillin-clavulanic acid, AMP: ampicillin, ATM: aztreonam; SAM: ampicillin-sulbactam, FEP: cefepime, FOX: cefoxitin, CAZ: ceftazidime, COL: colistin; CRO: ceftriaxone, CXM: cefuroxime, CIP: ciprofloxacin, CLI: Clindamycin, CTX: cefotaxime, ERY: erythromycin, ETP: ertapenem, GEN: gentamicin, IPM: imipenem, LNZ: linezolid, LVX: levofloxacin, MEM: meropenem, OXA: oxacillin, TCY: tetracycline, TGC: tigecycline, TZP: piperacillin-tazobactam, TOB: tobramycin, and SXT: trimethoprim-sulfamethoxazole, VAN: vancomycin. ND: no data. Comparison of antibiotic resistance between clinical specimens was performed using chi-square, or Fisher's exact test as appropriate. A two-tailed p-value <0.05 was considered statistically significant.
